# The risks of advancing parental age on neonatal morbidity and mortality are U- or J-shaped for both maternal and paternal ages

**DOI:** 10.1186/s12887-020-02341-0

**Published:** 2020-09-28

**Authors:** James A. Thompson

**Affiliations:** College of Veterinary Medicine and Biomedical Science, Texas A&M University, College Station, TX 77843-4475 USA

**Keywords:** Bayesian, Neonatal, Morbidity, Mortality, Maternal age, Paternal age

## Abstract

**Background:**

The biologic implications of delayed parenthood have been blamed for a major public health crisis in the United States, that includes high rates of neonatal morbidity and mortality (NMM). The objective of this study was to evaluate the risk of parent age on NMM and to provide results that can serve as a starting point for more specific mediation modeling.

**Methods:**

Data containing approximately 15,000,000 birth records were obtained from the United States Natality database for the years 2014 to 2018. A Bayesian modeling approach was used to estimate the both the total effect and the risk adjusted for confounding between parent ages and for mediation by chromosomal disorders including Down syndrome. Outcomes included intra-hospital death and nine measures of neonatal morbidity.

**Results:**

For paternal age, seven NMM (preterm birth, very preterm birth, low Apgar score, treatment with antibiotics, treatment with surfactant, prolonged ventilation, intra-hospital death) had U-shaped risk patterns, two NMM (small for gestational age, admission to neonatal intensive care) had J-shaped risk patterns, one NMM (seizures) was not significantly related to paternal age. For maternal age, three NMM (low Apgar score, treatment with antibiotics and intra-hospital death) had U-shaped risk patterns, four NMM (preterm delivery, very preterm delivery, admission to neonatal intensive care, treatment with surfactant) had J-shaped risk patterns, one NMM (small for gestational age) had a risk declining with age, one NMM (prolonged ventilation) had a risk increasing with age and one NMM (seizures) was not significantly related to maternal age.

**Conclusions:**

Both advancing maternal and paternal ages had U- or J-shaped risk patterns for neonatal morbidity and mortality.

## Background

For 50 years there has been a continuous increase in the age at which men and women, living in developed countries, are having children [1]. Many are exercising a choice to delay parenthood, largely in order to complete higher levels of education but also to establish employment and family stability [1, 2]. Educational attainment, job and financial security and father involvement all have positive health effects on the fetus and newborn [3–6]. However, the biologic implications of delayed parenthood have been blamed for a major public health crisis in the United States [7, 8]. Biologic risks have been largely attributed to gamete aging with meiotic non-disjunction among maturing oocytes [9] and accumulated mitotic errors among spermatogonia [10]. The disease burden attributable to chromosomal nondisjunction including Down syndrome is quite large [9]. However, the role of maternal age in causing neonatal morbidity and mortality (NMM) in the absence of maternal aneuploidy is controversial [11]. Similarly, for men, the empirical evidence that age-associated de novo mutations cause NMM has been controversial [8]. The genetic risks of advancing age on NMM are likely to be counteracted by risk reduction mediated by socioeconomic factors [12]. The net risk for each NMM is very likely non-linear and modeling age using broad age-categories will be inadequate for describing and interpreting this resultant risk function [13, 14]. More complex causal modeling is needed but will be challenged by cofounding between parent ages [15]. When modeling the joint effects of maternal and paternal ages, two approaches have predominated [16]. The linear or curvilinear (linear and quadratic) approach is usually inadequate because the functions will often fit well over specific age ranges and fit poorly over other age ranges. Dividing ages into categories has been preferred by many with 10-year age groups the most common approach but because of the very high correlation this approach is certain to leave residual confounding within categories [16]. Recently, it was shown that Bayesian modelling of joint maternal and paternal age effects with conditional autoregressive (CAR) priors provided a much superior fit for the risks of Down syndrome and other chromosomal disorders [15]. The objective of this study was to estimate the effects of parent age on NMM controlling for confounding between maternal and paternal age and to separate the mediating effect of chromosomal disorders, including Down syndrome.

## Methods

Data containing approximately 15,000,000 birth records were obtained from the United States Natality database for the years 2014 to 2018. In the United States, state laws require birth certificates to be completed for all births, and federal law mandates national collection and publication of births and other vital statistics data. The National Vital Statistics System, the federal compilation of these data, is the result of the cooperation between the National Center for Health Statistics and the states to provide access to statistical information from birth certificates. The fields retained for analysis included both parents’ ages, the presence of Down syndrome (DS) and chromosomal disorders other than Down syndrome (CD). Nine indicators of neonatal morbidity were retrieved from the birth records, including small for gestational age (SGA) defined as the lowest ten percentile of birth weights for each day of gestational length [17], preterm birth (PTB; birth at < 37 weeks gestation), very preterm birth (VPTB; birth at < 32 weeks gestation), low Apgar score (< 4), admission to a neonatal intensive care unit (NICU), three different treatments (Yes/no; antibiotics; surfactant; prolonged (> 6rs) ventilation) and the incidence of seizures. Intra-hospital death was defined as neonatal death that occurred before discharge from the hospital. The study was evaluated by the Texas A&M Institutional Review Board (IRB) and determined to be exempt from IRB review.

Odds ratios for neonatal morbidity and mortality by DS and CD were estimated as follows: Data were cross tabulated for each of i = 2 levels (present/not present) for k = 10 NMM. For each row in the table Y_ik_ was the count of cases, at birth, and n_i_, the count of births. The counts, Y_ik_ were modeled as independent Binomial distributions conditional on an unknown rate parameter (μ_ik_)

$$ {\mathrm{Y}}_{\mathrm{i}\mathrm{k}}\sim \mathrm{Binomial}\left({\upmu}_{\mathrm{i}\mathrm{k}},{\mathrm{n}}_{\mathrm{i}}\right) $$

The rate parameter was given a Uniform(0,1) prior. The odds ratios were estimated by converting the rate parameter to an odds and calculating the ratio of the odds:

$$ {\mathrm{OR}}_{\mathrm{k}}=\left[{\upmu}_{2,\mathrm{k}}/\left(1-{\upmu}_{2,\mathrm{k}}\right)\left]/\right[{\upmu}_{1,\mathrm{k}}/\left(1-{\upmu}_{1,\mathrm{k}}\right)\right] $$

In order to estimate the total effect for maternal age, case counts for each of k = 10 NMM were cross tabulated by i = 35 maternal ages (15 to 49 years) for each NMM. For each row in the table Y_ik_ was the count of cases, at birth, and n_i_, the count of births. The counts were modeled as independent Binomial distributions conditional on an unknown rate parameter (μ_ik_)

$$ {\mathrm{Y}}_{\mathrm{i}\mathrm{k}}\sim \mathrm{Binomial}\left({\upmu}_{\mathrm{i}\mathrm{k}},{\mathrm{n}}_{\mathrm{i}}\right) $$

The logit of the rate parameter was then modeled as a linear function of the overall intercept and a random effect for each maternal age.

$$ \mathrm{Logit}\left({\upmu}_{\mathrm{ik}}\right)={\upalpha}_{\mathrm{k}}+{\mathrm{maternal}}_{\mathrm{ik}} $$

The intercept was given a flat, improper prior. The maternal prior was a minimally informative CAR or random walk prior of length 35 (ages (i) = 15 to 49). The precision of the CAR prior was specified as Uniform (0,10) on the standard deviation scale.

In order to estimate the total effect for paternal age, case counts for each of k = 10 outcomes were cross tabulated by j = 51 paternal ages (15 to 65 years). For each row in the table, Y_jk_ was the count of cases, at birth, and n_j_, the count of births. The counts were modeled as independent Binomial distributions conditional on an unknown rate parameter (μ_jk_).

$$ {\mathrm{Y}}_{\mathrm{j}\mathrm{k}}\sim \mathrm{Binomial}\left({\upmu}_{\mathrm{j}\mathrm{k}},{\mathrm{n}}_{\mathrm{j}}\right) $$

The logit of the rate parameter was then modeled as a linear function of the overall intercept and a random effect for paternal age.

$$ \mathrm{Logit}\left({\upmu}_{\mathrm{jk}}\right)={\upalpha}_{\mathrm{k}}+{\mathrm{paternal}}_{\mathrm{jk}} $$

The intercept was given a flat, improper prior. The paternal prior was a minimally informative CAR or random walk prior of length 51 (ages (j) = 15 to 65). The precision of the CAR prior was specified as Uniform (0,10) on the standard deviation scale.

In order to estimate the adjusted effect for maternal age, data were restricted to neonates who were negative for DS and CD. The modeling of maternal age risk was adjusted for paternal age, as follows. Case counts for each of k = 10 outcomes were cross tabulated by i = 35 maternal ages (15 to 49 years) and j = 51 paternal ages (15 to 65 years). For each row in the table Y_ijk_ was the count of cases, at birth, and n_ij_, the count of births. The counts were modeled as independent Binomial distributions conditional on an unknown rate parameter (μ_ijk_).

$$ {\mathrm{Y}}_{\mathrm{ij}\mathrm{k}}\sim \mathrm{Binomial}\left({\upmu}_{\mathrm{ij}\mathrm{k}},{\mathrm{n}}_{\mathrm{ij}}\right) $$

The logit of the rate parameter was then modeled as a linear function of the overall intercept and a random effect for each maternal and paternal age.

$$ \mathrm{Logit}\left({\upmu}_{\mathrm{ijk}}\right)={\upalpha}_{\mathrm{k}}+{\mathrm{maternal}}_{\mathrm{ik}}+{\mathrm{paternal}}_{\mathrm{jk}} $$

The intercept was given a flat, improper prior. The maternal age prior was a minimally informative CAR or random walk prior of length 35 (ages (i) = 15 to 49) and the paternal age prior was a minimally informative CAR or random walk prior of length 51 (ages (j) = 15 to 65). The precision for the CAR priors was specified as Uniform (0,10) on the standard deviation scale.

In order to estimate the adjusted effect for paternal age, all data including data for neonates identified with DS and CD were used. The model was the same as used to estimate the maternal adjusted effect. All age-related odds ratios were standardized to age 15. The implementation used Markov Chain Monte Carlo (MCMC) and the software MultiBUGS 1.0.0 [18, 19]. All parameters were estimated with each iteration of the Markov Chain. Five thousand iterations were allowed for burn-in and each hundredth of the next 200,000 iterations were collected for the posterior distribution. Convergence was determined by observing multiple chains with disparate starting values. Bayesian credible intervals were taken directly from the full posterior distributions. Throughout this report, “adjusted” risk means that the risk of the parent’s age was adjusted for the risk of the other parent’s age.

A significant age effect was defined as at least one age having a Bayesian posterior predictive *p*-value of < 0.05 [20]. For the purpose of describing study results, a U-shaped distribution was defined as a distribution of age risks in which the risk for mid-range ages were significantly lower than both younger and older ages. A J-shaped distribution was defined as distribution of age risks in which mid-range age risks were significantly lower than younger ages and older ages were significantly higher than the youngest ages.

## Results

The accessed data identified 15,077,411 singleton births during the study period. Of these, 8323 mothers were less than 15y of age and 2492 were older than 49y. Among fathers, 67,096 were younger than 15 y and 3516 were older than 65 years. Father’s age was missing for 1,780,585 births which included births for which the father was not identified. There were 7913 births for which both the mother’s age was out of the study’s age range and the father’s age was missing or out of the study’s age range. It was very common for mothers aged less than 15y for the father’s age to be missing (72%; 6008/8323). In total, 1,862,012 births were deleted for missing or out of range parent ages. Outcome variables were missing for 14,701 births and these births were deleted. In total, 13,207,486 births were used in the analyses.

**Fig. 1:**
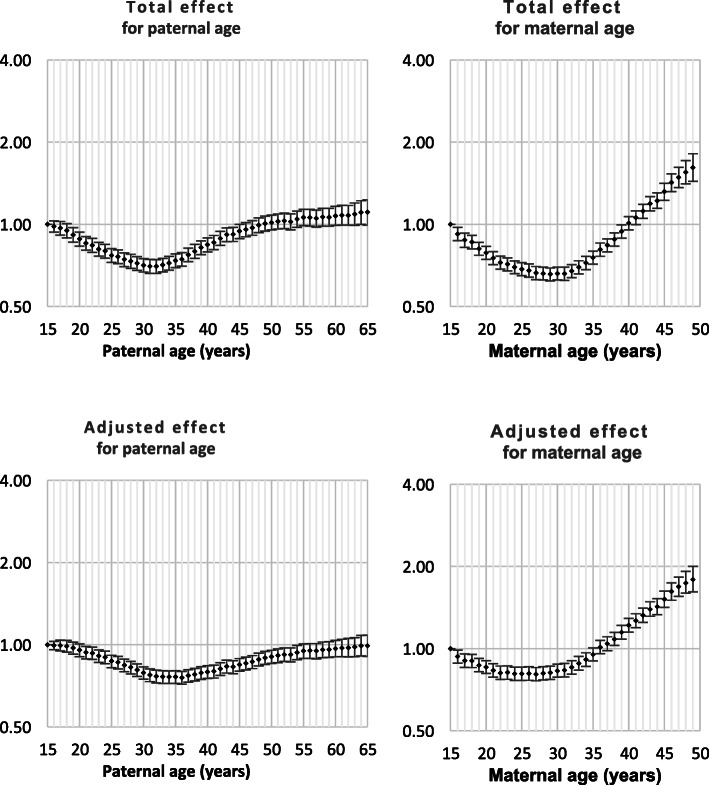
Odds ratios for total and adjusted effects for preterm birth by parent age relative to age 15 years

**Fig. 2:**
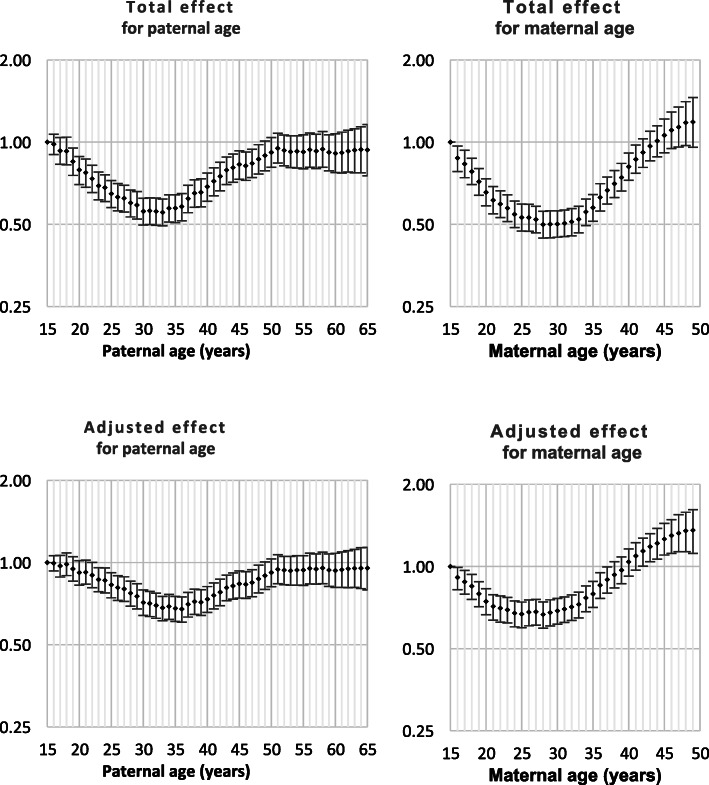
Odds ratios for total and adjusted effects for very preterm birth by parent age relative to age 15 years

**Fig. 3:**
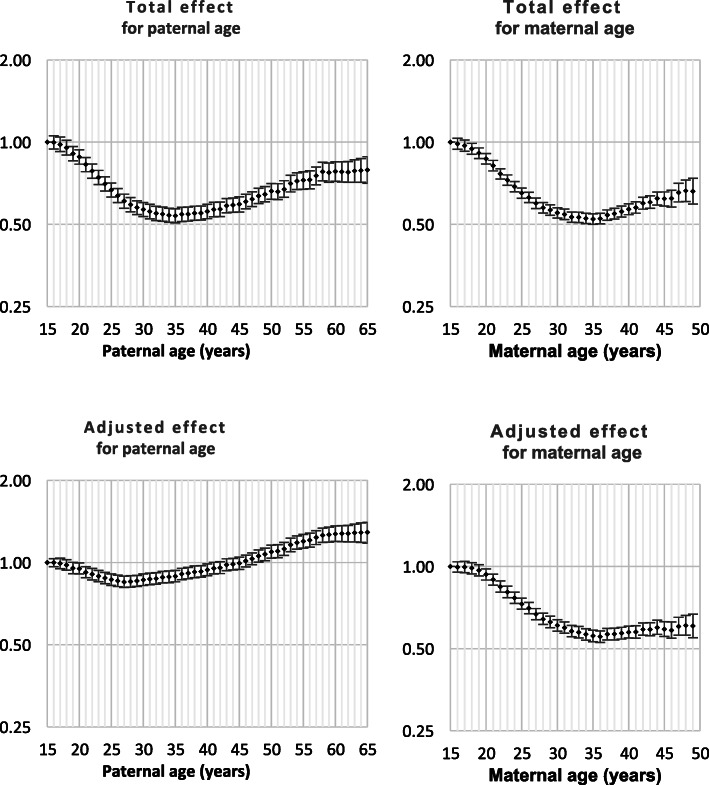
Odds ratios for total and adjusted effects for small for gestational age by parent age relative to age 15 years

**Fig. 4:**
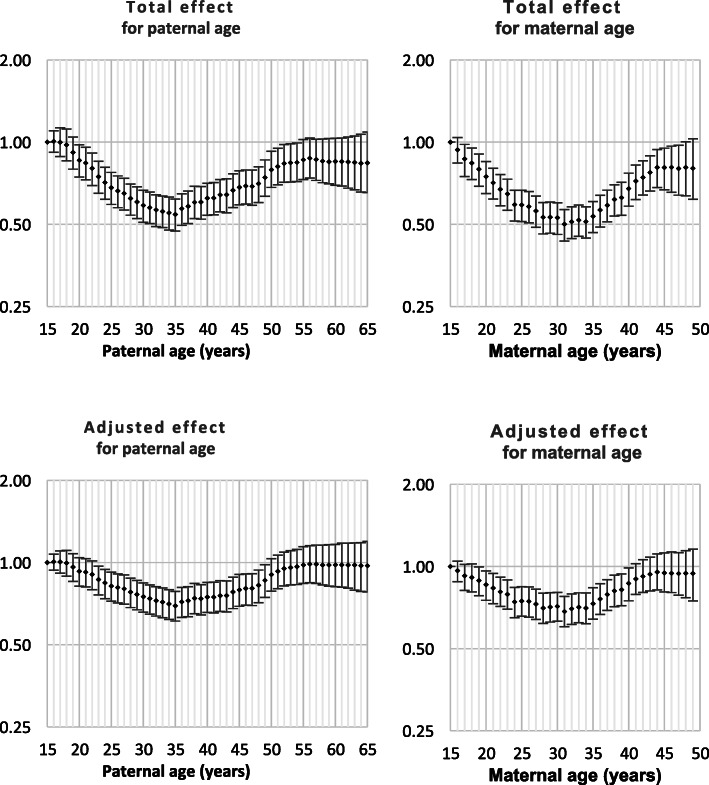
Odds ratios for total and adjusted effects for low Apgar score by parent age relative to age 15 years

**Fig. 5:**
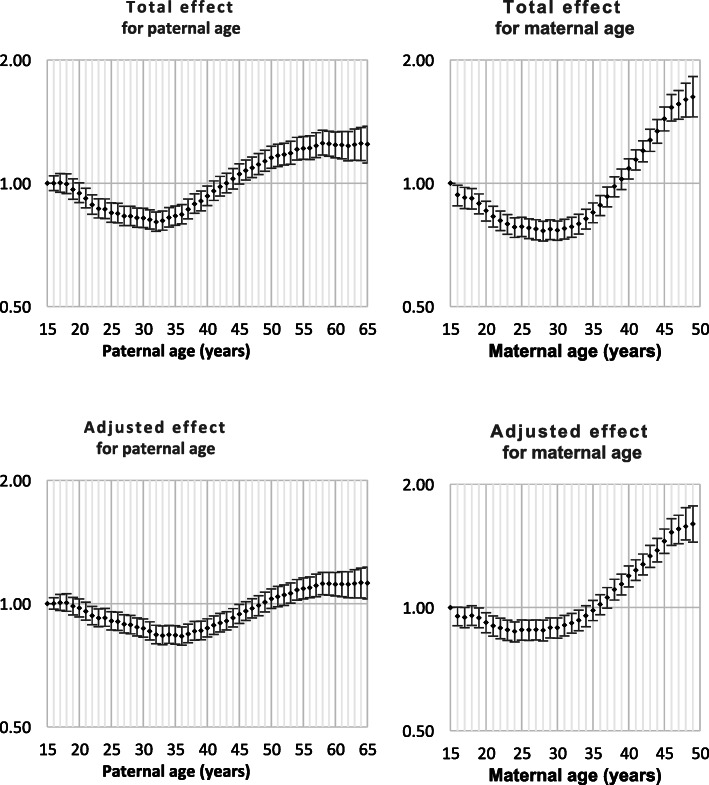
Odds ratios for total and adjusted effects for admission to neonatal intensive care unit by parent age relative to age 15 years

**Fig. 6:**
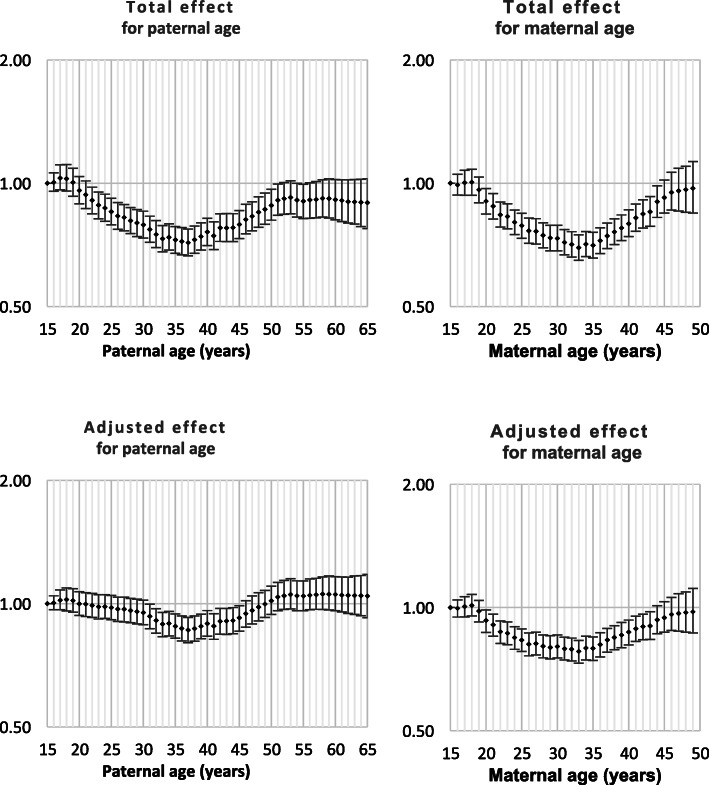
Odds ratios for total and adjusted effects for treatment with antibiotics by parent age relative to age 15 years

**Fig. 7:**
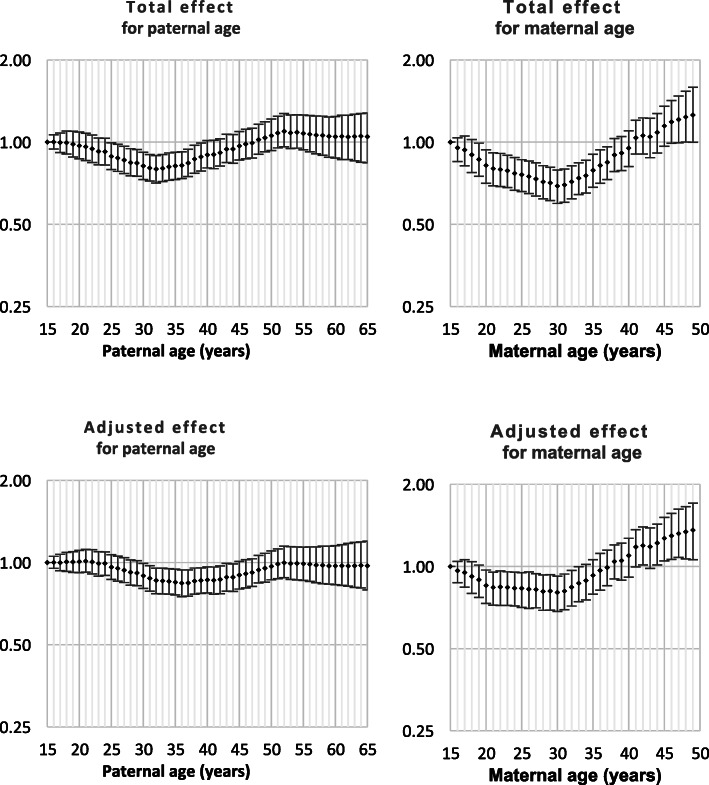
Odds ratios for total and adjusted effects for treatment with surfactant by parent age relative to age 15 years

**Fig. 8:**
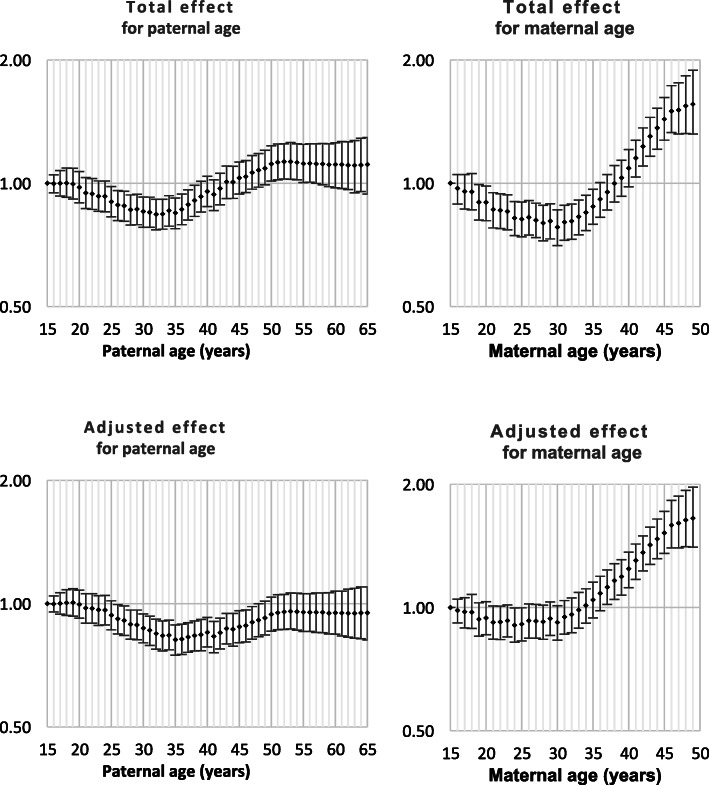
Odds ratios for total and adjusted effects for treatment with prolonged ventilation by parent age relative to age 15 years

**Fig. 9:**
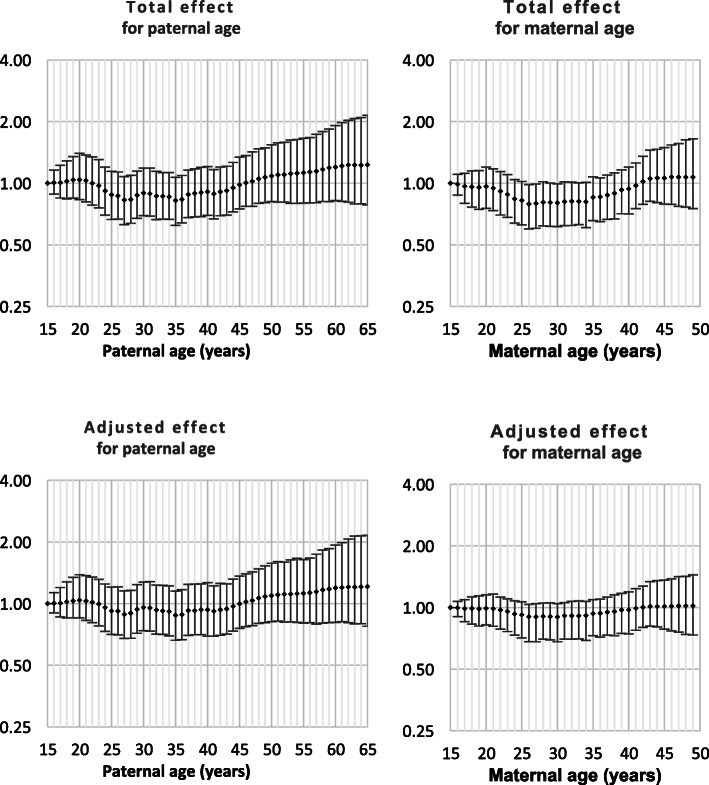
Odds ratios for total and adjusted effects for occurrence of seizures by parent age relative to age 15 years

**Fig. 10:**
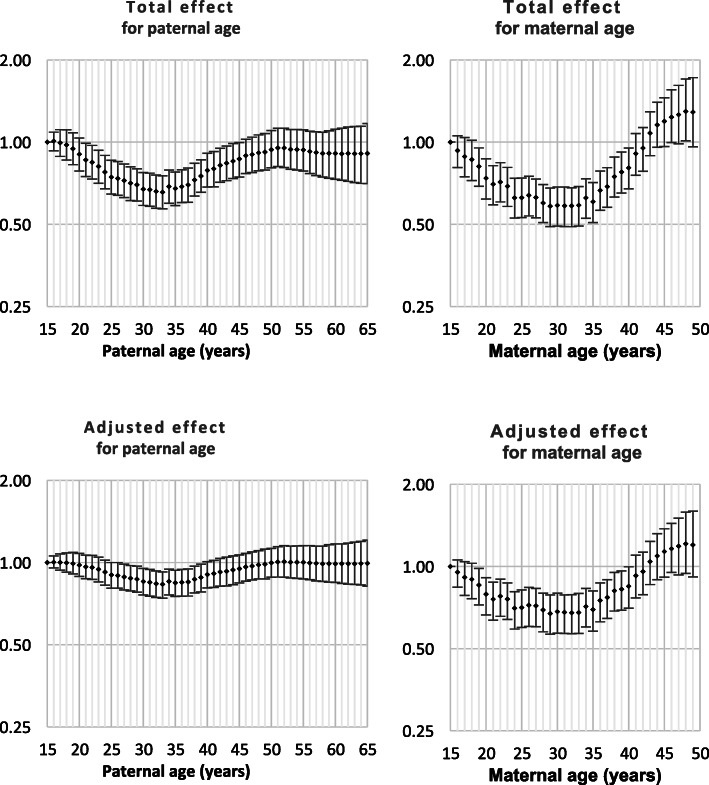
Odds ratios for total and adjusted effects for neonatal death by parent age relative to age 15 years

Both Down syndrome and chromosomal disorder (other than Down syndrome) were strongly related to intra-hospital death and all nine monitored morbidities (Table 1). Odds ratios by parent age for both total effect and adjusted effect are presented in Figs. 1, 2, 3, 4, 5, 6, 7, 8, 9, 10. The figures show that age 15y was not the lowest risk age for either maternal or paternal age. For maternal age, the lowest risk was more commonly age 30y and for paternal age 35y. The adjusted risks for outcomes among NMM from children born to the observed extreme ages relative to maternal age 30 y and paternal age 35y are presented in Table 2. For paternal age, seven NMM (preterm birth, very preterm birth, low Apgar score, treatment with antibiotics, treatment with surfactant, prolonged ventilation, intra-hospital death) had U-shaped risk patterns, two NMM (small for gestational age, admission to neonatal intensive care) had J-shaped risk patterns, one NMM (seizures) was not significantly related to paternal age. For maternal age, three NMM (low Apgar score, treatment with antibiotics and intra-hospital death) had U-shaped risk patterns, four NMM (preterm delivery, very preterm delivery, admission to neonatal intensive care, treatment with surfactant) had J-shaped risk patterns, one NMM (small for gestational age) had a risk declining with age, one NMM (prolonged ventilation) had a risk increasing with age and one NMM (seizures) was not significantly related to maternal age.

**Table 1:** Odds ratios for neonatal morbidity and mortality for neonates born with Down syndrome or chromosomal disorders other than Down syndrome

	Odds ratio for Down syndrome	Odds ratio for chromosomal disorders^1^
Preterm Delivery	4.2 (4.0, 4.4)	6.0 (5.6, 6.4)
Very preterm delivery	2.8 (2.4, 3,2)	8.1 (7.3, 9.0)
Small for gestational age	2.3 (2.2, 2.4)	5.2 (4.9, 5.5)
Low Apgar score	4.7 (4.0, 5.6)	31.4 (28.7, 34.1)
Admission to NICU	15.7 (15.0, 16.5)	15.9 (15.0, 16.8)
Antibiotics	7.8 (7.3, 8.3)	11.4 (10.6, 12.3)
Surfactant	6.0 (5.0, 7.1)	16.4 (14.4, 18.6)
Prolonged ventilation	12.7 (11.7, 13.7)	22.9 (21.3, 24.6)
Seizure(s)	11.7 (7.7, 17.0)	49.7 (39.0, 62.5)
Death	12.6 (11.1, 14.3)	70.2 (65.0, 75.7)

^1^ Chromosomal disorders other than Down syndrome

**Table 2:** Odds ratios for youngest and oldest parents relative to age 30y for mothers and age 35y for fathers

	Odds ratio for paternal age			Odds ratio for maternal age		
	Age 15y	Age 30y	Age 65y	Age 15y	Age 35y	Age 49y
Preterm delivery	1.31 (1.25, 1.39)	1 (baseline)	1.30 (1.20, 1.40)	1.22 (1.16, 1.30)	1 (baseline)	2.20 (2.00, 2.44)
Very preterm delivery	1.45 (1.31, 1.62)	1	1.39 (1.19, 1.62)	1.47 (1.32, 1.65)	1	2.00 (1.70, 2.35)
Small for gestational age	1.13 (1.07, 1.19)	1	1.46(1.35, 1.57)	1.60 (1.53, 1.67)	1	0.96 (0.88, 1.06)
Low Apgar score	1.42 (1.26, 1.62)	1	1.38 (1.16, 1.66)	1.41 (1.25, 1.61)	1	1.32 (1.09, 1.57)
Admission to NICU	1.19 (1.13, 1.25)	1	1.34 (1.24, 1.44)	1.12 (1.06, 1.18)	1	1.80 (1.64, 1.96)
Antibiotics	1.12 (1.05, 1.20)	1	1.17 (1.05, 1.30)	1.25 (1.18, 1.34)	1	1.22 (1.10, 1.38)
Surfactant	1.17 (1.05, 1.31)	1	1.14 (0.95, 1.36)	1.23 (1.08, 1.45)	1	1.67 (1.38, 2.12)
Prolonged ventilation	1.20 (1.10, 1.30)	1	1.13 (1.00, 1.29)	1.06 (0.97, 1.17)	1	1.76 (1.53, 2.06)
Seizure(s)	1.09 (0.82, 1.44)	1	1.32 (0.93, 2.27)	1.11 (0.94, 1.45)	1	1.13 (0.91, 1.62)
Death	1.17 (1.06, 1.31)	1	1.16 (1.00, 1.38)	1.48 (1.27, 1.77)	1	1.79 (1.42, 2.30)

## Discussion

The current study evaluated the risk of both parents’ ages on individual NMM that were observed in the very early neonatal period and recorded in the national collection of birth records. Multiple NMM often occur together and there has been considerable interest in using composite scores derived from multiple NMM. However, there has been no consistency in the implementation of composite outcomes and the general approach has, thus far, proven to be too heterogenous [21]. As an alternative, the current study could be followed up with a Bayesian approach in which NMM are modeled as a multivariate distribution and the correlations among the NMM are objectively estimated. This approach could be used to evaluate specific programs (elements of prenatal care, for example) as preventive for multiple correlated outcomes. The treatments administered would be important components of these sets of correlated events. Treatments are administered more subjectively and are influenced by both subjective evaluation by clinicians and institutional guidelines. Despite this subjectivity, treatments were significantly related to both parents’ ages from both statistical and clinical perspectives.

The current study adds considerable precision and detail to what is known about the risks of both parents’ ages on NMM. The precision results from autoregressive smoothing [22]. When the data are highly correlated, at 1-year age intervals, the gain in precision is large and when there is no evidence of correlation at 1-year intervals, the risk estimates are the same as in the model that assumes independence among age groups. Modeling of 1-year age groups, as independent, has been published and showed similar trends but considerably less precision [13].

There is existing evidence of a U-shaped risk distribution for maternal age [13]. In the Weng study, age 27y was used as the baseline low risk definition. At maternal age 16y odds ratios for preterm birth, small for gestational age and neonatal death were 2.03 (1.88, 2.20), 1.83 (1.68, 1.99) and 2.33 (1.76, 3.08), respectively and for age > 43y were 2.62 (2.31, 2.97), 0.95 (0.92, 0.97) and 2.65 (1.46, 4.61), respectively. The current study showed that the maternal age risk for eight NMM declined from age 15y to age 30y. For four outcomes, the maternal age risk at advanced ages was significantly higher than at very young ages. It may be more correct to call these five risk functions J-shaped rather than U-shaped. A recent meta-analysis showed that the most common analysis of maternal age effects used 10-year age categories which has limitations in describing long-term trends that are non-linear. However, the meta-analysis confirmed that advanced maternal age was related to preterm delivery, higher rates of NICU and worse Apgar scores [14].

The existing support for U-shaped risk function for paternal age is limited. One study used 10-year age ranges and age 25-34y as standard. The odds ratios for preterm delivery, low Apgar scores and admission to NICU were 1.15 (1.15, 1.16), 1.23 (1.23, 1.16) and 1.03 (1.03, 1.04), respectively for age < 25y and were 1.65 (1.62, 1.69), 134 (1.29, 1.39) and 1.64 (1.59, 1.68), respectively for age > 55y. Literature review showed that comparison of broad age categories is the most common comparison and that these comparisons provided little or no support for any difference among paternal ages for preterm birth, small for gestational age and neonatal death [8]. The current study showed that paternal age risks declined from age 15y to age 35y for nine of ten outcomes. For all nine of these NNM, the paternal age risk increased from age 35y. For two NMM, the risk of advanced age was significantly higher than young paternal ages and the risk function could be called J-shaped. The odds ratios for the extreme paternal ages were small compared to the oldest and youngest maternal age odds ratios.

**Fig. 11:**
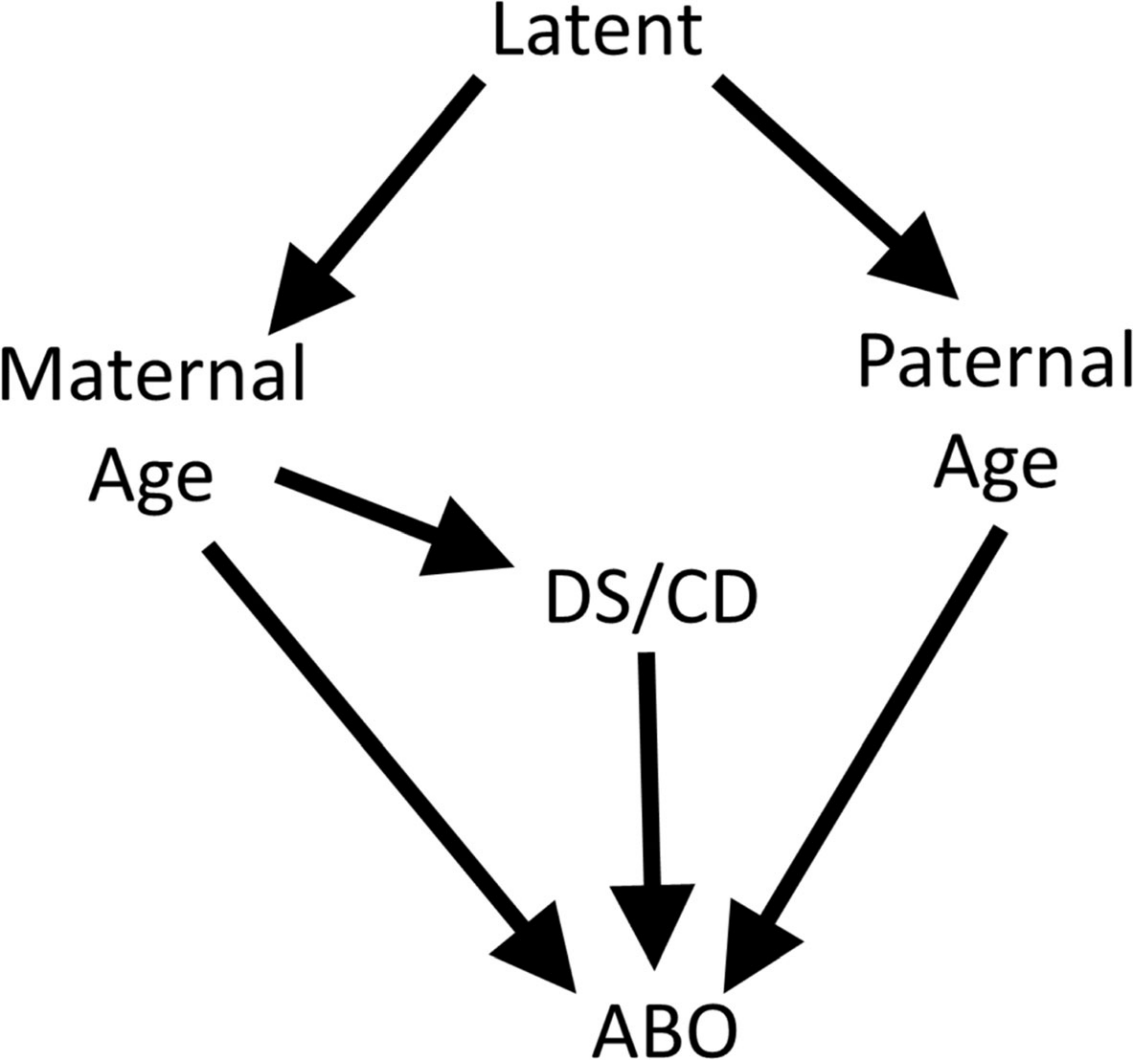
The Directed Acyclic Graph (DAG) used in the study

The current study took a novel approach to the management of covariates for the purpose of very specific objectives aimed at facilitating mediation modeling. The first step was to evaluate the unadjusted effects of both maternal and paternal age allowing for the effect to be non-linear. Graphical evaluation of the Total Effect, as defined in mediation modeling [23–25], confirmed the existence of parental age risks that were non-linear. The adjusted effects were estimated accounting for potential confounding between maternal and paternal age which are known to be highly correlated [16]. The current study confirmed that the ages of both parents have independent effects on NMM. Further mediation analysis will need to control for the confounding between parent ages and must account for effects that are not linear. We believe that the joint CAR method for age modeling will be the best possible option to control this confounding in further mediation modeling. Maternal age effects were estimated including the effect of chromosomal disorders, including Down syndrome (CD/DS) as a potential mediator. The results show that there exists a significant disease burden that is not mediated by CD/DS. A directed acyclic graph (DAG; Fig. 11) was used to guide this analysis [26]. The graph shows that one or more variables cause maternal and paternal ages to be similar. To block confounding between paternal and maternal ages, it would be theoretically possible to block the backdoor path by blocking this variable, if it were known. As an alternative, blocking by maternal age for paternal age and by paternal age for maternal age provides the same control of confounding. Blocking paternal age by maternal age also prevents confounding of the paternal age risk by chromosomal disorders including Down syndrome (CD/DS). A previous study on the same population showed that there was no direct effect between paternal age and CD/DS so CD/DS could confound the relationship but does not mediate paternal age effects [15]. These explicit conditions provide a structural approach for investigators to estimate alternative estimates that would be considered “causal” by improving upon the current DAG [26, 27]. A better understanding of the biology that constitutes the observed non-linear net aging effect should be pursued by developing more specific causal models. The current study adds to the existing literature by providing a specific causal model that can, and will be, criticized for its omission of important causes including both confounders and mediators.

## Conclusions

Both advancing maternal and paternal ages had U- or J-shaped risk patterns for neonatal morbidity and mortality.

## Data Availability

The database used in the study is publicly available and requires users to agree to a Data Use Agreement (https://www.cdc.gov/nchs/data_access/vitalstatsonline.htm).

## Abbreviations

CD: Chromosomal disorders, excluding Down syndrome
CD/DS: Chromosomal disorders, including Down syndrome
DAG: Directed acyclic graph
DS: Down syndrome
IRB: Institutional Review Board
MCMC: Markov Chain Monte Carlo
NICU: Neonatal Intensive Care Unit
NMM: Neonatal morbidity and mortality
PTB: Preterm birth (birth prior to 37 weeks gestation)
SGA: Small for gestational age (smallest 10 percentile for gestational age)
VPTD: Very preterm birth (birth prior to 32 weeks gestation)
